# Wound healing complications after tooth extraction in dogs

**DOI:** 10.3389/fvets.2026.1962193

**Published:** 2026-10-21

**Authors:** Luka Šparaš, Matic Pavlica, Barbara Lukanc, Ana Nemec

**Affiliations:** Small Animal Clinic, Veterinary Faculty, University of Ljubljana, Ljubljana, Slovenia

**Keywords:** dogs (*Canis familiaris*), exodontia, small animal dentistry, wound dehiscence, wound healing complications

## Abstract

**Objective:**

This prospective clinical study aimed to assess incidence of wound healing complications associated with extraction sites following permanent tooth extractions in healthy dogs.

**Materials and methods:**

Eighty-seven client-owned healthy adult dogs undergoing clinically indicated closed or open dental extractions, comprising 404 extraction sites, were included in the study. No perioperative and immediate postoperative antibiotics or antiseptics were used in any of the dogs. All dogs were discharged on the day of the procedure with pain management as clinically indicated and underwent a routine (non-sedated) follow-up examination 10 days later. Sites with incomplete epithelial healing and wound edge separation were classified as dehisced. Weekly in-person or telemedicine follow-up examinations were performed until complete healing was confirmed.

**Results:**

Dehiscence was the most common postoperative complication, diagnosed in 19.5% (17/87) of the dogs and 4.2% (17/404) of the extraction sites within 10 days after the procedure. Most affected sites involved large, functionally important teeth, and 76.5% (13/17) of cases were mild. None of the dehisced sites required surgical revision, and only 23.5% (4/17) dogs required additional medical management with antibiotics and/or analgesia. All dehisced sites healed by second intention within one month. Increasing body weight was associated with higher odds of dehiscence, with a 13% increase in odds for each additional kilogram of body weight. Removal of at least 50% of the alveolar bone, extraction in the rostral mandibular region, and the use of triangular flaps during open extraction were also significantly associated with increased odds of dehiscence. Traumatic dentoalveolar injury was the only indication for tooth extraction significantly associated with increased odds of dehiscence.

**Conclusions and clinical relevance:**

Extraction wound dehiscence was uncommon at the individual-site level, but remained a clinically relevant postoperative complication when assessed per treated dog. Most affected sites healed uneventfully by second intention within one month, without surgical revision or prolonged medical management. This finding supports conservative management of extraction wound dehiscence in uncomplicated cases.

## 1 Introduction

Exodontia is one of the most frequently performed procedures in veterinary dental practice. Indications for tooth extraction in dogs include periodontal disease (1), endodontal disease such as dental fractures (2), tooth resorption (3), and other conditions such as malocclusion (4, 5) or odontodysplasia (6). Healing of a dental extraction wound in dogs is a well-described process involving a series of complex biological events, including formation and remodelling of soft and bony tissues (7–9). Typically, the healing of the extraction site is clinically completed within two to 4 weeks and demonstrates specialized healing by second intention (7, 9). Primary closure of the extraction wound facilitates retention of the blood clot within the alveolus (which is an essential component of post-extraction healing), while also protects the extraction site from debris accumulation (10). Studies in various mammalian models have shown that rapid wound healing with minimal or no scarring is a fundamental characteristic of oral mucosal tissues, primarily because oral mucosa differs from skin in both structure and physiology (11). Dehiscence following exodontia is reported as one of the most commonly encountered complications in small animal dentistry (12, 13), leading to delayed wound healing and possibly alveolar osteitis (14). A previous clinical study in dogs showed that endodontal and periodontal pathologies may cause changes to the alveolar bone leading to delayed extraction socket healing (7). Despite the high frequency of dental extractions in veterinary dental practice there is a lack of detailed literature exploring dehiscence and other extraction wound healing complications, including incidence rates and possible contributing factors in veterinary dentistry (10, 12, 13, 15–17). Pegg et al. (13) reported that the traditionally recommended simple-interrupted suture pattern was associated with greater debris accumulation and inflammation of the adjacent tissues. The same study also observed a higher rate of suture loss with the simple-interrupted pattern compared with the simple-continuous pattern, although this difference was not statistically significant. This gap in the veterinary literature leaves many clinicians relying on personal experience or anecdotal evidence to manage healing complications after tooth extractions. Additionally, the approach to treating these complications can vary among practitioners, with no universally accepted standardized protocol. In humans, wound-healing complications after tooth extraction are well recognized, although their reported frequency varies widely among studies (18–22). Their management depends on the extent of tissue loss, presence of infection, and patient’s overall healing capacity (22–24).

The primary aim of this prospective clinical study was to evaluate the incidence of wound healing complications associated with extraction sites following permanent tooth extractions in systemically healthy dogs. Additionally, we investigated potential contributing factors to wound healing complications after tooth extraction.

## 2 Materials and methods

### 2.1 Study population

Client-owned adult dogs with permanent dentition, scheduled for dental procedures requiring tooth extraction at the Small Animal Clinic, Veterinary Faculty, University of Ljubljana, Slovenia, were considered to be included in this study over a 12-month period. To be included in the study, the dogs had to be systemically healthy as per clinical examination, complete blood count (CBC) and serum biochemistry (with a minimum requirement of alanine aminotransferase (ALT), creatinine (CREA), and urea (BUN) and additional tests conducted based on the dog’s age and clinical history). Dogs classified as American Society of Anaesthesiologists (ASA) status II with mild systemic abnormalities but without clinically relevant functional impairment (e.g., grade I/VI heart murmurs, age >10 years, or mild dermatologic conditions not requiring medication) were also included in the study. Dogs with underlying medical conditions or receiving treatments (e.g., immunomodulatory medication) that could impair wound healing were excluded from the study. Exclusion criteria also included the need for perioperative or postoperative antibiotic therapy and the presence of an oronasal fistula in any dog included in the study. In addition, dogs that did not allow non-sedated oral examination and dogs, whose owners declined an in-person follow-up examination, were also excluded from the study. All dog owners signed an informed consent form for the procedures and the use of the anonymised data. This study was approved by the Animal Welfare Committee at the Veterinary Faculty, University of Ljubljana, Slovenia (approval number 033-5/2025-38).

### 2.2 General anaesthesia

Food was withheld for no more than 12 h before anaesthesia, and water was allowed ad libitum until the procedure. The dogs were premedicated with dexmedetomidine (Dexdomitor, Zoetis, United States) 2 μg/kg and methadone (Comfortan, Dechra, United Kingdom) 0.2 mg/kg intramuscularly (IM). After an intravenous (IV) catheter was placed, anaesthesia was induced with propofol (Propomitor, OrionPharma, Finland) titrated to effect until endotracheal intubation. Anaesthesia was maintained with isoflurane (Isofane, Covetrus, United Kingdom) in oxygen and air (FiO2 = 0.4) delivered through a circle breathing system (22 mm Flextube Breathing System, Intersurgical LTD, United Kingdom). The dogs breathed spontaneously during anaesthesia. They were positioned in dorsal recumbency on a dentistry table covered with soft padding. After induction, oral nerve blocks (infraorbital and/or inferior alveolar) were performed with a fine 42 mm 27-gauge needle (Sulzer Mixpac GmbH, Germany) using 0.5% levobupivacaine (Chirocaine, AbbVie, United States) as clinically indicated with a cumulative dose of levobupivacaine not exceeding 2 mg/kg per animal. For both blocks, the bevel of the needle was always oriented in the same direction as the nerve fibers, and negative pressure was always applied to the syringe before drug injection. All blocks were performed aseptically by a board-certified veterinary dentist and/or a resident. Systolic arterial pressure (SAP) was measured noninvasively with an ultrasonic Doppler flow monitor (Model 811-BL, Parks Medical Electronics, United States) every 5 min. If hypotension occurred (SAP below 80 mmHg), a bolus of 10 mL/kg isotonic crystalloid solution was administered IV over 10 min. Heart rate, respiratory rate, end-tidal isoflurane concentration, end-tidal CO2 tension, and ECG (lead II) were monitored continuously with a multiparameter monitor (B105 Patient Monitor, GE Medical Systems Information Technologies Inc., United States) and recorded every 5 min. Body temperature was measured every 15 min with a rectal thermometer, and forced-air warming (Bair Hugger, 3 M, United States) was used when temperature decreased below 37 °C. Ringer’s lactate solution (B Braun Medical Inc., United States) at 5 mL/kg/h was administered via infusion pump (Infusion Pump SK-600I Vet, Shenzhen Mindray Scientific Co. LTD, China) during the procedure. Carprofen (Rycarfa, Krka, Slovenia) 4 mg/kg IV was given to all dogs when SAP was at least 80 mmHg. Ketamine (Ketamidor, Richter Pharma AG, Austria) 1 mg/kg IV was administered as a rescue analgesic when heart rate or SAP increased by more than 20% from baseline values. Propofol 1 mg/kg IV was administered, and the isoflurane setting was increased by 0.1% when a positive palpebral reflex was observed to deepen anaesthesia. Duration of general anaesthesia was defined as the time from anaesthetic induction to extubation.

### 2.3 Diagnostic and surgical protocol

Once the dog was anaesthetized and placed in dorsal recumbency, a complete oral and dental examination, including detailed dental charting and full-mouth dental radiographs, was performed by a board-certified veterinary dentist and/or a resident. Dental extractions were performed as clinically indicated, following routine operating protocols ensuring aseptic approach (10, 15–17, 25). Periodontal treatment was always performed before any dental extractions. No perioperative antibiotics nor antiseptics were used. The decision regarding the extraction procedure (closed versus open) was based on the type of tooth and clinical and dental radiographic findings. Open extractions were performed using a full-thickness mucoperiosteal extended envelope, triangular, or pedicle flap, and this was documented. Sulcular and releasing incisions were made with a #15 scalpel blade on a round scalpel handle no. 5. If multiple adjacent teeth required extraction, a single flap was used and documented as one extraction site with multiple teeth extracted. Bone removal, if necessary, was performed using an appropriately sized round carbide bur on a high-speed handpiece with water irrigation, with the amount of labial or buccal bone removed (as a percentage of root length) documented. If alveolar bone was lost due to advanced periodontitis, the amount of bone removal was documented as percentage of the remaining alveolar bone. Teeth were extracted using luxators and extraction forceps. Osteoplasty was performed with a round diamond bur on a high-speed handpiece with water irrigation. Vacated alveoli were gently debrided with a periosteal elevator and a blood clot was allowed to fill in the alveolus before suturing the soft tissues. All extraction sites were sutured in a simple-interrupted pattern using 5–0 poliglecaprone 25 suture material with a reverse-cutting needle. In open extractions, care was taken to achieve tension-free closure; in closed extractions, sutures were placed only to approximate the wound edges and prevent displacement of the blood clot. Any intraoperative complications were recorded, and all extraction sites were routinely radiographed upon completion of the extraction. All procedures were performed by a board-certified veterinary dentist and/or a resident under supervision, and all procedures and any complications were routinely recorded in the dental chart and dogs’s electronic record. Extraction indications were categorized as periodontal disease (stage 3 or 4; the decision to extract teeth with stage 3 periodontal disease depended on the extent of alveolar bone loss and whether the owner declined advanced periodontal treatment and/or was unable to provide regular oral home care (26)), complicated fracture (of any subtype, as classified according to AVDC nomenclature (27)), other pathology (i.e., teeth with radiographic evidence of endodontal disease without concurrent fracture and teeth with advanced tooth resorption), or combined pathologies (i.e., extraction sites involving multiple teeth with differing pathologies). Extraction sites were also classified by anatomic location. Sites were designated as incisive region when the extracted teeth were incisor teeth of either the maxilla or mandible. The rostral maxillary region was defined as extending from the maxillary canine tooth to the third premolar tooth, whereas the caudal maxillary region extended from the fourth premolar to the second molar tooth. In the mandible, the rostral mandibular region extended from the canine tooth to the third premolar tooth, and the caudal mandibular region extended from the fourth premolar tooth to the third molar tooth. Extraction sites involving both rostral and caudal portions of the jaw were classified based on the most rostral aspect of the sulcular incision of the flap.

### 2.4 Postoperative management and follow-up

After the procedure, methadone 0.2 mg/kg IV was administered and repeated every 4 hours as clinically indicated. The dogs were prescribed oral carprofen (Rimadyl, Pfizer, United States) 2 mg/kg every 12 h for 2–7 days. When severe pain was anticipated, a transdermal fentanyl patch (Durogesic, Janssen, United States) at 4 μg/kg/h was applied. After the procedure, Ringer’s lactate solution was administered at 2 mL/kg/h until discharge of the dog home. All dogs were discharged home the same evening. Owners received detailed dietary recommendations (soft food only for 7–10 days), oral care guidelines (temporary cessation of tooth brushing), and instructions to avoid hard food and chew toys until at least the first follow-up visit. No topical antiseptics were used. Clients were also advised about signs of potential postoperative complications, including halitosis, marked facial swelling, pain during mastication or inappetence, oral discharge, or bleeding from the surgical site. A routine in-person follow-up examination of the non-sedated animal was scheduled 10 days after the procedure and was conducted by one of the surgeons who had performed the procedure. If any complications were reported by the owner, a recheck appointment was scheduled sooner to determine the need for further intervention. At this re-check examination, an extraction site was classified as dehisced when epithelial healing was incomplete and the site showed wound edge separation, defects of varying extent, and granulation tissue with associated signs of inflammation. The severity of wound dehiscence was numerically graded using an adapted wound dehiscence scoring system previously described by Pegg et al. (13) (Table 1). The scoring system was adapted for examination of non-sedated animals; the presence of inflammation, debris, necrotic tissue, discharge, or ulceration was not assessed because these features could not be evaluated consistently. If any extraction wound-healing complications were identified at the initial follow-up examination, weekly in-person or telemedicine rechecks, including the submission of high-quality photographs, were scheduled until complete clinical healing of the wound was confirmed.

**Table 1:** Wound dehiscence scoring system adapted by Pegg et al. (13).

Dehiscence score	Comparison
0	Complete epithelial healing at the mucosal edges. No separation or areas of granulation tissue.
1	Incomplete epithelial healing, gaps <10 mm bridged by granulation tissue.
2	Incomplete epithelial healing, gaps >10 mm bridged by granulation tissue.
3	Incomplete epithelial healing, gaps >10 mm not bridged by granulation tissue.

### 2.5 Statistical analysis

Statistical analysis was performed in R version 4.3.3. Continuous variables are presented as median and interquartile range (IQR), and categorical variables as counts and percentages. Associations with dehiscence were assessed at the extraction-site level using logistic regression models. Because multiple extraction sites could originate from the same dog, all analyses accounted for within-dog clustering. Random-intercept mixed-effects logistic regression was fitted using glmer() from the lme4 package when model fitting was stable and the dog-level variance was estimable (28). When sparse data led to unstable mixed-model fits, singularity, convergence problems, or other indications of unreliable random-effects estimation, logistic regression was fitted using glm(), and inference was based on dog-level cluster-robust standard errors using the sandwich and lmtest packages (29–31). Owing to the low number of dehiscence events, models were kept parsimonious and generally included a single predictor or a limited set of biologically related predictors. Results from frequentist models are reported as odds ratios (OR) with 95% confidence intervals (CI). *Post hoc* pairwise comparisons for multi-level categorical predictors were performed using the emmeans package, with Tukey adjustment for multiple comparisons (32).

For flap type, conventional maximum-likelihood mixed-effects logistic regression was unsuitable because sparse cell counts and complete separation resulted in unstable parameter estimation. A Bayesian mixed-effects logistic regression was therefore fitted using stan_glmer() from the rstanarm package (33), allowing the dog-level random intercept to be retained while obtaining finite, regularised estimates. The reference category was none and the model used a logit link. Because flap type was structurally linked to extraction type in this dataset, an additional Bayesian sensitivity analysis was performed within the subset of open extractions only; in this model, Envelope was used as the reference category. The same specifications and settings were used for both Bayesian flap models, such that the open-only analysis differed only in the restricted dataset and the chosen reference category. Weakly informative priors were specified as follows: fixed-effect coefficients were assigned Normal (0, 0.5) priors with autoscaling enabled, the intercept was assigned a Normal (−3.1, 1) prior on the log-odds scale with autoscaling disabled, and the random-intercept covariance prior was decov (regularization = 2). Sampling used 4 chains with 4,000 iterations per chain, including 2000 warmup iterations, yielding 8,000 post-warmup draws. Convergence, sampling diagnostics, model fit, and posterior predictive checks were assessed. Bayesian results are reported as posterior median odds ratios (OR) with 95% credible intervals (CrI), while posterior probabilities for the direction of effect are provided in the Supplementary material. Limited sensitivity analyses were performed for selected associations by adding one clinically relevant covariate.

## 3 Results

### 3.1 Study population

87 healthy adult dogs were included in this study. 28 dogs had an ASA status I and 59 dogs ASA status II. The median age was 8 years (IQR: 5–10; range, 1–15), and the median body weight was 12.8 kg (IQR: 7.3–22.1; range, 2.0–45.6). Of these dogs, 41 were spayed females (47.1%), 21 were castrated males (24.1%), 15 were intact males (17.2%), and 10 were intact females (11.5%). Breeds of the dogs varied greatly with 20 of the dogs being mix breed and 67 dogs belonging to 43 different breeds (with 1–4 dogs of the same breed).

### 3.2 General anaesthesia

The median duration of anaesthesia was 120 min (IQR: 100.0–152.5; range, 60–240), and the median lowest recorded temperature was 36.6 °C (IQR: 36.1–37.3; range, 33.9–39.0). Five animals (5.2%) received an intravenous bolus of isotonic crystalloid solution due to SAP < 80 mmHg.

### 3.3 Indications for extraction and surgical procedures

In this study 404 extraction sites (including 507 extracted teeth) were evaluated. The majority of extractions sites were located in the incisive region of both jaws (188/404; 46.5%), followed by the caudal maxilla (84/404; 20.8%), rostral maxilla (73/404; 18.1%), caudal mandible (34/404; 8.4%), and rostral mandible (25/404; 6.2%). The median number of extraction sites per dog was 3 (IQR: 1.0–6.0; range, 1–16).

Closed dental extractions were performed in 54.5% (220/404) of extraction sites, while open dental extractions accounted for 45.5% (184/404).

For open extractions, the triangular flap was most commonly used (129/184; 70.1%), followed by the extended envelope flap (50/184; 27.2%) and the pedicle flap (5/184; 2.7%). In open extractions, 51.6% involved removal of at least 50% of the alveolar buccal bone, whereas in 48.4% of cases, alveolectomy involved less than 50% bone removal. Flap type was associated with the extent of bone removal (Fisher’s exact test, *p* < 0.001); ≥50% bone removal occurred in 79/129 (61.2%) triangular, 5/50 (10.0%) envelope, and 3/5 (60.0%) pedicle flap cases (Supplementary Table S4A).

Advanced periodontal disease was the predominant indication for dental extraction, accounting for 78.7% (318/404) of extraction sites. Traumatic dentoalveolar injuries represented 13.6% (55/404) of extraction sites and involved 58 teeth, including complicated crown fractures (37/58; 63.8%), complicated crown-root fractures (19/58; 32.8%), and root fractures (2/58; 3.4%). These injuries most commonly affected the maxillary fourth premolar teeth (24/58; 41.4%), followed by the incisor teeth (13/58; 22.4%) and canine teeth (9/58; 15.5%). Other pathologic conditions were indication for extraction in 4.2% (17/404) of sites, including teeth with radiographic evidence of endodontal disease without concurrent fracture (82.4%) and teeth affected by tooth resorption (17.6%). Extraction sites involving multiple teeth with differing underlying pathologies were categorized as combined pathologies and accounted for 3.5% (14/404) of sites.

### 3.4 Surgery-related complications and management

The only intraoperative complication encountered was iatrogenic fracture of the tooth root, which occurred in 9.9% (40/404) of extraction sites and 7.9% (40/507) of all extracted teeth in 27.6% (24/87) of treated dogs. In all cases, at least 75% of the alveolar bone was subsequently removed, the fractured roots were successfully retrieved, and dental radiography confirmed complete evacuation of the alveoli. All 40 extraction sites with an intraoperative root fracture were classified in the ≥50% bone-removal category. A detailed cross-tabulation of root fracture, bone removal, and dehiscence is provided in Supplementary Table S4B. Dehiscence was the most common postoperative complication and was recorded in 17 of 87 dogs (19.5%) and at 17 of 404 extraction sites (4.2%). Most extraction sites complicated by dehiscence involved larger, functionally important teeth, particularly canine teeth (6/17; 35.3%), maxillary fourth premolar teeth (4/17; 23.5%), and mandibular first molar teeth (1/17; 5.9%), together accounting for 64.7% (11/17) of affected sites. Most (76.5%) dehisced extraction sites were classified as mild, with dehiscence scores of 1 (Figure 1) or 2 (Figure 2). In the remaining sites, the dehisced areas were not bridged by granulation tissue at the first postoperative examination [dehiscence score of 3 (Figure 3)] (13). Antibiotic therapy was initiated 5 days after the procedure in three of 17 cases with dehiscence. In one case, the owner, a practicing veterinarian, initiated treatment independently, where we would deem it unnecessary (dehiscence score of 2). In the other two cases, the dehisced sites involved mandibular canine extraction sites and were assigned a dehiscence score of 3. The owners of these two dogs also reported marked halitosis and pain with eating. In all three cases, amoxicillin–clavulanic acid was prescribed at 15–17.5 mg/kg twice daily for 5 days. Carprofen administration was extended for an additional three to 5 days in these cases. The same analgesic regimen was also prolonged in one additional dog with a dehiscence score of 2 after the owner reported pain during eating. In all cases of dehiscence, postoperative home care measures were continued until the affected site had healed. These included feeding soft food, avoiding tooth brushing at the surgical site, and preventing access to hard chew toys. Owners were also instructed to gently rinse the affected area with saline using a syringe after meals. All dehisced sites were clinically healed within 31 days after the procedure without any revision surgery (Figures 2, 3).

**Figure 1:**
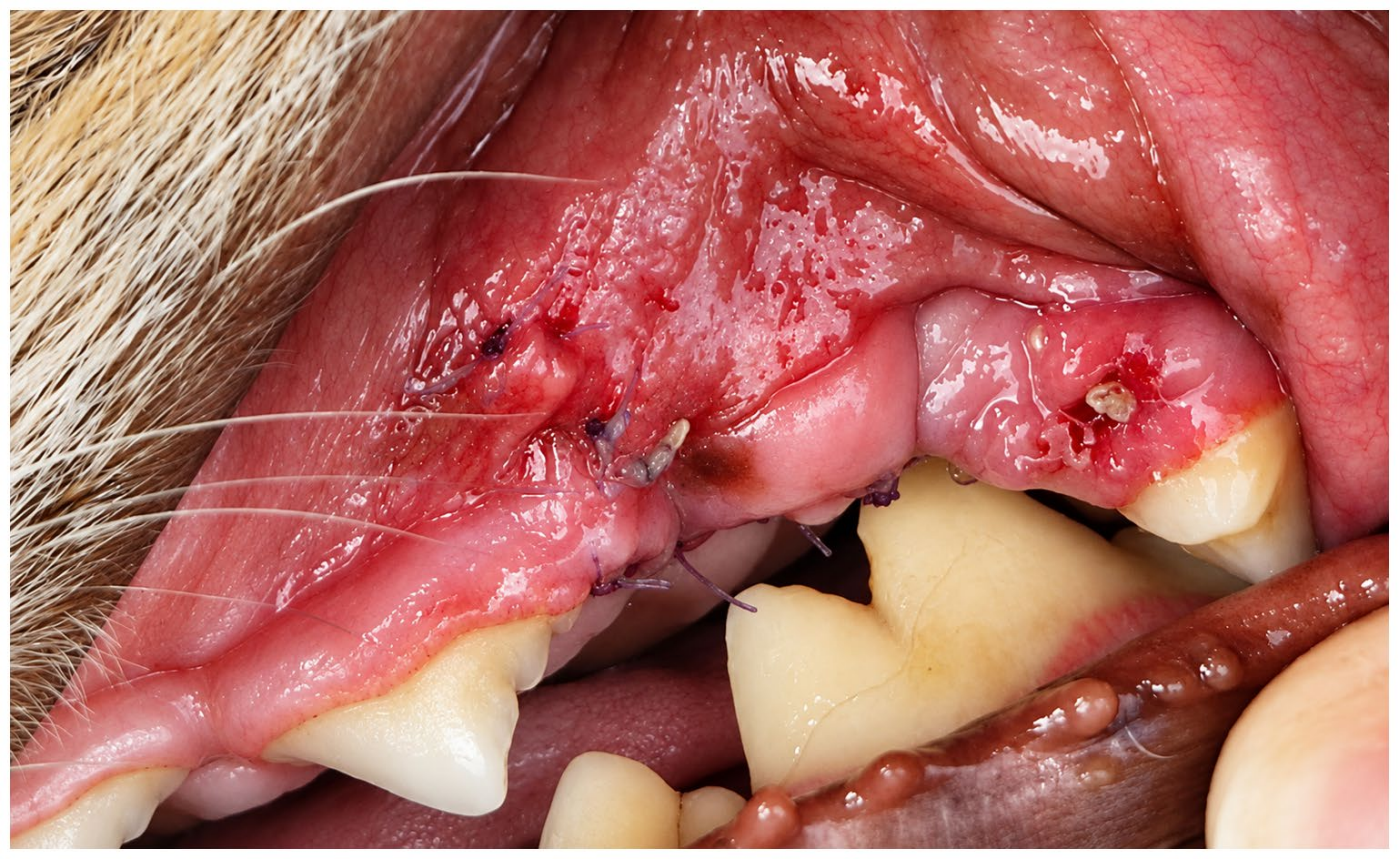
Representative image showing a wound dehiscence score of 1 at 10 days after an open (triangular flap) extraction of the left maxillary fourth premolar tooth in a 21-kg dog due to complicated crown fracture. Incomplete epithelial healing with a small gap bridged by granulation tissues is noted (black arrows) during the clinical recheck.

**Figure 2:**
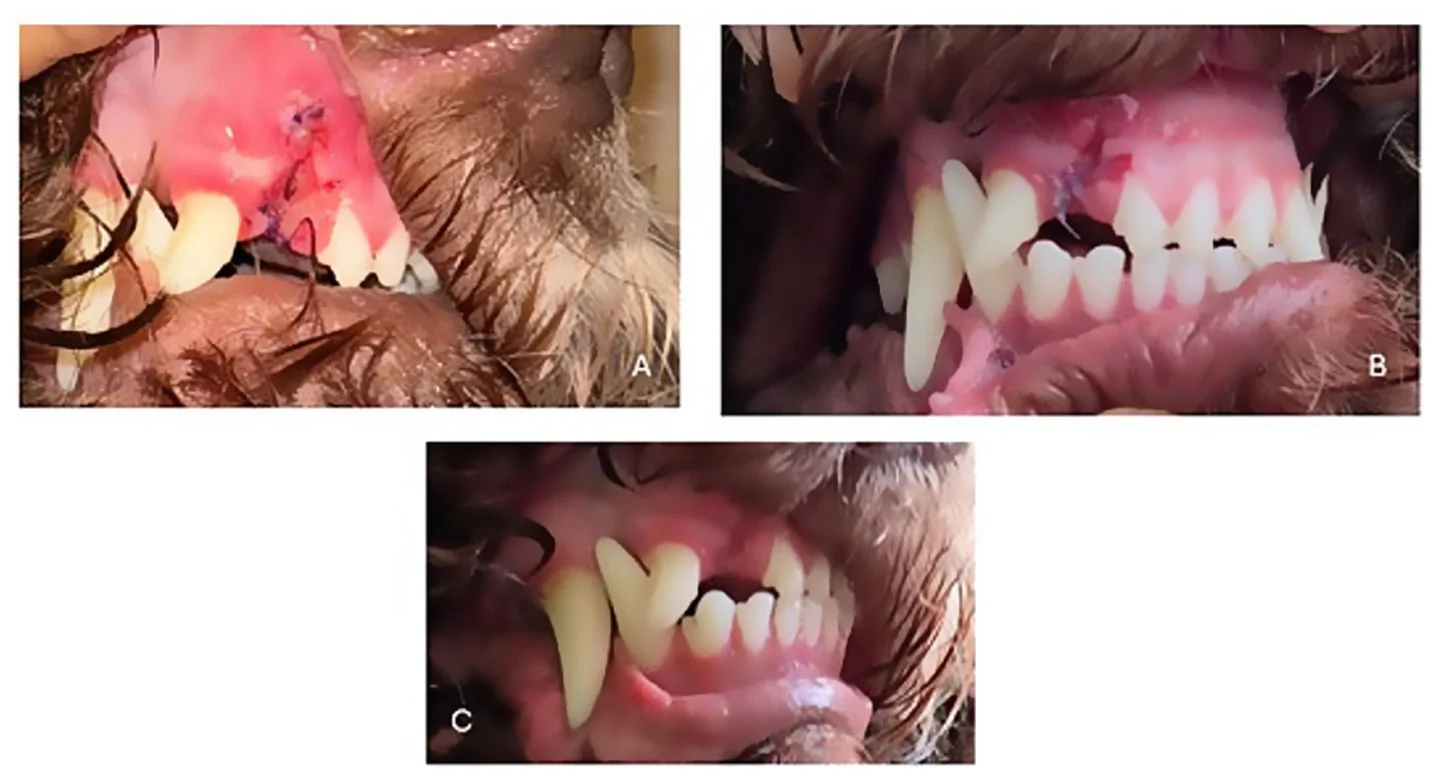
Representative images showing a wound dehiscence score of 2 and the subsequent healing process after an open extraction (triangular flap) of the right second maxillary incisor tooth in a 13-kg dog due to complicated crown fracture. **(A)** Appearance of the extraction wound 3 days after the procedure, following the owner’s report of clinical signs suggestive of impaired healing. The same wound 13 **(B)** and 21 **(C)** days after the procedure.

**Figure 3:**
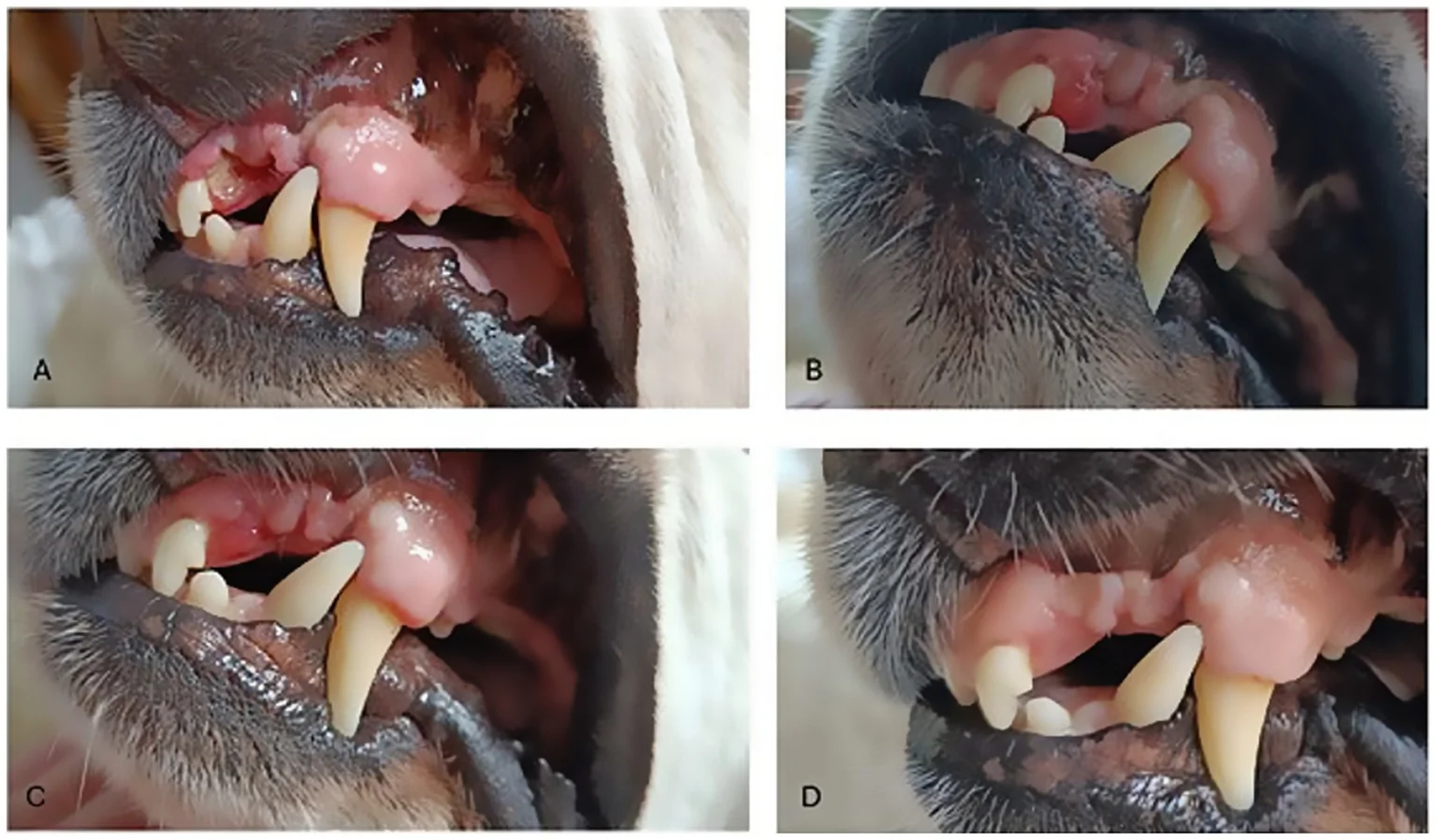
Representative images showing a wound dehiscence score of 3 and the subsequent healing process after a closed extraction of the left third maxillary incisor tooth in a 26-kg dog due to complicated crown fracture. **(A)** Appearance 3 days after the procedure, following the owner’s report of clinical signs suggestive of impaired healing. Note the incomplete epithelial healing with a large gap not bridged by granulation tissue, exposing some underlying alveolar bone. The same wound 10 **(B)**, 17 **(C)** and 23 **(D)** days after the procedure, when complete healing was confirmed without any surgical or medical intervention.

Other rare postoperative complications included suborbital soft tissue swelling in two dogs, ipsilateral to the extracted teeth. In both cases, the swelling developed more than 14 days after the procedure, and the extraction wounds had healed uneventfully at the first postoperative examination. Head CT was performed in both dogs for further diagnostic evaluation. In one dog, an unidentified foreign body was detected subcutaneously and removed, with no recurrence of swelling observed thereafter. In the second dog, CT revealed soft tissue swelling, and biopsy findings were consistent with panniculitis and myositis. The swelling resolved following 2 weeks of treatment with amoxicillin–clavulanic acid (17.5 mg/kg twice daily), selected based on bacterial culture and antimicrobial susceptibility testing.

No complications related to local anaesthetic administration were recorded in any of the 87 treated dogs.

### 3.5 Factors associated with dehiscence

#### 3.5.1 Age and weight

Age of the dog was not associated with dehiscence (*p* = 0.826). In contrast, body weight was positively associated with dehiscence (*p* = 0.003), indicating that each 1 kg increase in body weight was associated with a 13% increase in the odds of dehiscence (Table 2).

**Table 2:** Summary of mixed-effects logistic regression models for dehiscence.

Predictor	Comparison/unit	Events	OR	95% CI	*p*
Age	per 1 year		1.03	0.80–1.32	0.826
Body weight	per 1 kg		1.13	1.04–1.23	0.003
Extraction type	Closed (reference)	1/220			
	Open	16/184	17.31	2.11–141.91	0.008

Events are presented as number of dehiscence events/total extraction sites in each category. OR = odds ratio; CI = confidence interval. Mixed models include a random intercept for dog.

#### 3.5.2 Anatomical factors

Anatomical region was associated with dehiscence overall (*p* = 0.003). Compared with the incisive region, the rostral mandible showed higher odds of dehiscence (*p* < 0.001), corresponding to a 23.25-fold increase in odds (Table 3). The rostral maxilla was also associated with higher odds of dehiscence compared with the incisive region (*p* = 0.027; Table 3). In contrast, the caudal mandible and caudal maxilla were not significantly associated with dehiscence compared with the incisive region. In Tukey-adjusted *post hoc* comparisons, only the contrast between the rostral mandible and incisive region remained significant (adjusted *p* = 0.004; Supplementary Table S1), and no other pairwise differences were observed.

**Table 3:** Summary of clustered logistic regression models for dehiscence.

Predictor	Comparison/unit	Events	OR	95% CI	*p*
Anatomical site	Incisive region (reference)	2/188			
	Caudal mandible	1/34	2.82	0.24–33.50	0.412
	Caudal maxilla	4/84	4.65	0.80–27.08	0.087
	Rostral mandible	5/25	23.25	4.03–134.27	<0.001
	Rostral maxilla	5/73	6.84	1.24–37.64	0.027
Reason for extraction	Periodontal (reference)	4/318			
	Fracture	10/55	9.20	2.84–29.75	<0.001
	Combined	1/14	2.05	0.21–20.06	0.538
	Other	2/17	6.87	0.82–57.91	0.076
Extent of bone removal	None (reference)	1/211			
	<50%	2/98	2.51	0.23–27.91	0.454
	≥50%	14/95	18.92	2.36–151.59	0.006
Intra-op complication	Absent (reference)	13/364			
	Present	4/40	3.00	0.83–10.78	0.092
Hypotension	Absent (reference)	15/381			
	Present	2/23	2.32	0.48–11.33	0.297
Anaesthesia duration	per 10 min		0.90	0.79–1.03	0.125
Lowest temperature	per 1°C		2.10	1.18–3.75	0.011

Events are presented as number of dehiscence events/total extraction sites in each category. OR = odds ratio; CI = confidence interval. Models use cluster-robust standard errors clustered by dog.

#### 3.5.3 Surgical factors

The model, which included reason for extraction and extent of buccal bone removal, was associated with dehiscence overall (*p* < 0.001). Extractions performed because of traumatic dentoalveolar injuries had significantly higher odds of dehiscence than those performed because of periodontal disease, with an approximately 9-fold increase in odds (*p* < 0.001; Table 3). In contrast, extractions due to other pathologies or combined pathologies were not significantly associated with dehiscence.

For the extent of buccal bone removal, removal of at least 50% was associated with higher odds of dehiscence compared with no buccal bone removal (*p* = 0.006), corresponding to a 19-fold increase in odds (Table 3). In contrast, buccal bone removal of less than 50% was not significantly associated with dehiscence. In Tukey-adjusted *post hoc* comparisons, the ≥50% buccal bone removal category differed significantly from both no buccal bone removal (adjusted *p* = 0.016) and <50% buccal bone removal (adjusted *p* = 0.031; Supplementary Table S2).

Extraction type was also associated with dehiscence. In a mixed-effects logistic regression model, open extraction was associated with higher odds of dehiscence than closed extraction (*p* = 0.008), corresponding to a nearly 17-fold increase in the odds of dehiscence (Table 2).

In the Bayesian logistic regression model, flap type was associated with differences in dehiscence risk. Compared with closed extraction, the triangle flap showed a clear increase in the odds of dehiscence. Although the pedicle flap also showed higher estimated odds, this finding was uncertain because of the wide credible interval. By contrast, the envelope flap showed no clear association with dehiscence. In pairwise comparisons, only the triangle flap differed clearly from the envelope flap, whereas the comparison between the pedicle and envelope flaps remained uncertain because of sparse data (Table 4). Because flap type was structurally linked to extraction type in this dataset, an additional Bayesian sensitivity analysis was performed within open extractions only (Table 4). In the analysis restricted to open extractions, both the triangle and pedicle flaps showed higher estimated odds of dehiscence than the envelope flap, although the estimate for the pedicle flap was substantially more uncertain because of the wide credible interval (Table 4). No clear difference was detected between the triangle and pedicle flaps (Supplementary Table S3).

**Table 4:** Summary of the Bayesian logistic regression model of dehiscence by flap type.

Predictor	Comparison	Events	OR	95% CrI	Posterior prob.
Flap type (full sample)	None (reference)	1/220			
	Envelope	0/50	0.45	0.04–3.52	*p* (OR > 1) = 0.238
	Pedicle	1/5	13.30	0.43–172.00	*p* (OR > 1) = 0.940
	Triangle	15/129	8.56	3.08–26.70	*p* (OR > 1) > 0.999
Open extractions only	Envelope (reference)	0/50			
	Pedicle	1/5	6.19	0.24–79.40	*p* (OR > 1) = 0.878
	Triangle	15/129	4.44	1.18–20.00	*p* (OR > 1) = 0.986

Events are presented as number of dehiscence events/total extraction sites in each category. OR = odds ratio; CrI = equal-tailed credible interval. Posterior probability is the proportion of posterior draws with OR > 1.

Sites with intraoperative complications showed higher estimated odds of dehiscence than those without complications, but this association was not statistically significant (*p* = 0.092; Table 3).

#### 3.5.4 Perioperative factors

Anaesthesia duration was not associated with dehiscence (*p* = 0.125), nor was hypotension (*p* = 0.297; Table 3). In contrast, minimum intraoperative temperature was associated with dehiscence, with higher recorded minimum temperatures corresponding to a 2.10-fold increase in odds per 1 °C increase (*p* = 0.011; Table 3). However, this finding should be interpreted cautiously, as the association was attenuated and no longer statistically significant after adjustment for body weight (*p* = 0.183), suggesting possible confounding.

## 4 Discussion

To the authors’ knowledge, this is to date the largest prospective clinical study to evaluate the incidence of post-extraction healing complications in healthy adult dogs. In addition to providing relevant clinical data, this study may also serve as a baseline to more studies investigating approaches to improve extraction wound healing.

Wound dehiscence is defined as the breakdown or reopening of a surgical wound or suture line (34, 35). To clinically evaluate this in the present study, dogs were routinely examined 10 days after the procedure, since wound dehiscence in veterinary patients typically occurs during the early postoperative period, most often within the first week after surgery (36, 37). In dogs whose owners reported clinical signs suggestive of healing complications, such as halitosis, persistent oral discharge, trismus, pain during mastication, or marked facial swelling, the recheck examination was performed before the scheduled 10-day follow-up, most commonly this was indeed needed four to 5 days after the procedure. Clear discharge instructions regarding the clinical signs associated with healing complications after dental extractions are important for the owners to recognize possible adverse outcomes. This was in this study highlighted by the fact, that all dogs that were presented for an earlier recheck were confirmed to have some degree of dehiscence. In the absence of clinical signs, the timing of the first postoperative examination appeared clinically appropriate, as most dehisced extraction sites already showed granulation tissue formation and did not require further treatment. Earlier postoperative examination may have indeed found higher dehiscence rate and may have also identified less mature wound healing, potentially leading to different clinical interpretation or management. Conversely, a later first examination may have underestimated mild wound-healing disturbances, as some sites may already have healed by that time (13). When no signs of dehiscence or other healing complications were detected at the first recheck, no further examination was performed, as Pegg et al. (13) reported that dogs with uncomplicated healing 7 days after exodontia did not subsequently develop wound dehiscence. However, appropriate client counselling regarding continuous postoperative monitoring is warranted as some dogs may develop problems – related or not to the extraction procedure itself – also later, as observed in two dogs with suborbital swelling in this study.

Wound dehiscence occurred in 4.2% of extraction sites and in 19.5% of dogs included in the study. Although the site-specific prevalence was relatively low, the proportion of affected dogs highlights the clinical importance of this complication and the need for its recognition during case management and owner communication. However, the per-dog incidence should be interpreted with caution, as dogs with a greater number of extraction sites had more opportunities to develop dehiscence. Published human studies, primarily focused on surgical extraction of mandibular third molar teeth, have reported wound dehiscence rates ranging from approximately 11 to 68%, depending on the surgical approach and closure technique used (18–22). In dogs, reports on wound dehiscence rates after tooth extraction are much scarcer. A recent study comparing closed and open extraction techniques for deciduous canine teeth reported no cases of wound dehiscence following extraction (4). In contrast, a study in Greyhounds reported a much higher incidence of wound dehiscence, with at least some degree of dehiscence observed one week after the procedure in 68.4% of sites closed with a simple interrupted suture pattern and 68.8% of sites closed with a simple continuous suture pattern. However, by the second recheck, performed 14 days after the procedure, the proportion of dehisced wounds had decreased to 52.6 and 31.3%, respectively (13). Most dehisced extractions sites in the present study were classified as mild. As all recheck examinations in our study were performed in non-sedated dogs, only the size of the dehiscence and the presence of granulation tissue could be evaluated reliably, possibly underestimating the incidence of mild healing complications, such as individual suture failure, and no data on bone healing can be provided.

None of the dehisced sites required surgical revision and only 4 of 17 dogs were considered in need of additional medical (antibiotic and/or analgesia) management. Overall, most dehisced extraction sites (76.5%) healed uneventfully by second intention within a month without the need for local antiseptic and/or systemic antibiotic therapy and/or prolonged analgesic treatment. These findings are consistent with a previous study evaluating secondary wound healing after extraction of the maxillary first molar tooth in dogs where uneventful healing was reported in 126 of 127 surgical sites, and surgical revision was performed in only one site with impaired wound healing (38). Together, these data show that wound healing complications after properly performed dental extractions in healthy adult dogs without oronasal fistulae are relatively rare and mostly mild, and generally only require monitoring and reassurance of the clients.

In addition to determining the incidence of dehiscence, in this study we also evaluated factors associated with its occurrence. Increasing body weight was positively associated with extraction wound dehiscence with each kilogram increase in body weight associated with a 13% increase in the odds of dehiscence. One possible explanation is that larger dogs are more frequently presented with complicated dental fractures (39) that were also positively associated with dehiscence in this study. This association may reflect greater surgical difficulty of extracting fractured teeth in larger dogs and associated greater iatrogenic trauma. Namely, fractured teeth in larger dogs are often periodontally healthy, requiring more extensive alveolar bone removal to facilitate extraction (16). This hypothesis is further supported by the finding that removal of at least 50% of alveolar bone was also associated with increased odds of dehiscence. Findings from a human study assessing bone formation within the alveolus and contour changes of the alveolar process following tooth extraction suggested that the degree of crestal bone regeneration depends mainly on the existing bone level at the extraction site, rather than on the height of the bone surrounding adjacent teeth (40). Similarly, in human lower third molar surgery, deeper residual bone cavities after extraction have been associated with poorer early wound healing, whereas cavity width was not significantly related to healing (41). More recently, a study in dogs demonstrated that placement of an intra-alveolar regenerative adhesive biomaterial following mandibular tooth extraction resulted in improved preservation of alveolar margin dimensions immediately after the procedure. This effect was maintained during long-term follow-up, with greater preservation of residual alveolar margin dimensions still evident 12 months after extraction (42). The increased risk observed in the present study is unlikely to be explained by the extent of alveolar bone removal alone. Rather, greater bone removal likely indicates extractions where periodontal attachment is more intact and the procedure is therefore more challenging and traumatic. Opposite to this—greater bone removal was also performed in cases with intraoperative iatrogenic root fracture, which occurred in 9.9% of extraction sites, but this complication was not statistically significantly associated with a higher overall dehiscence rate.

The most common dental pathology treated by exodontia in this study was advanced periodontal disease as also reported before (43). This finding is unsurprising, given the high prevalence of periodontal disease in the canine population (1, 44, 45). Traumatic dentoalveolar injury was the second most common indication for exodontia in this study. Complicated crown fractures were the most frequent type of traumatic injury, consistent with previous reports (2, 46), although a recent study identified root fracture as the most common traumatic injury necessitating exodontia (43). Most traumatic injuries affected strategic teeth (canine and carnassial teeth), in agreement with earlier studies (46). The maxillary fourth premolar tooth was the most commonly affected, likely due to its important role in mastication and exposure to large forces (47, 48). Of all pathological categories assessed, traumatic dentoalveolar injury was the only one significantly associated with increased odds of dehiscence after exodontia. This supports the hypothesis that extraction of periodontally healthy teeth results in greater surgical trauma to the surrounding tissues and therefore a higher risk of wound dehiscence.

The rostral mandible (defined as the region extending from the mandibular canine to the third premolar tooth) was the only anatomical region significantly associated with dehiscence after Tukey adjustment for multiple pairwise comparisons. Mandibular canine tooth extraction sites were previously reported to have a higher dehiscence rate (13). However, in the present study, the increased risk observed in the rostral mandible is unlikely to be explained solely by greater surgical trauma associated with extraction of larger, non-periodontally affected teeth, as only two cases involved mandibular canine with crown fractures. Instead, the association may reflect regional anatomical and biomechanical features. The lower lip and perioral region are exposed to persistent dynamic forces generated by the perioral muscles, and wound tension is a well-recognized risk factor for dehiscence (13, 49–52). Therefore, increased local tension may partly explain the higher dehiscence rate observed in this region. Although the rostral maxilla (defined as the region extending from the maxillary canine to the third premolar tooth) showed higher odds of dehiscence compared with the incisive region in the main model, this difference did not remain significant after correction for multiple comparisons and should therefore be interpreted with caution.

For open extractions, triangular flaps were associated with a higher occurrence of dehiscence, whereas envelope flaps appeared to have the lowest risk. This contrasts with some human studies on mandibular third molar extraction, in which envelope flaps were more commonly associated with wound dehiscence than modified triangular or bayonet flaps (53–55). Pedicle flaps also appeared to be associated with increased risk in the present study, although this estimate was less precise. These findings should be interpreted cautiously, as flap selection was closely related to extraction difficulty. Flap selection was related to the extent of bone removal. Triangular flaps were more commonly used in extractions involving ≥50% bone removal, whereas envelope flaps were predominantly used when <50% of bone was removed. Therefore, the higher risk observed with triangular flaps may not be attributable to flap design alone. Although the additional releasing incision may increase the area susceptible to dehiscence, the association more likely reflects the greater difficulty and invasiveness of the extractions in which triangular flaps were used.

No intraoperative factor evaluated in this study was associated with increased odds of dehiscence, including intraoperative iatrogenic root fracture, duration of anaesthesia, and hypotension requiring an isotonic crystalloid fluid bolus. Root fracture during extraction attempts is a common complication (12, 56, 57). In the present study, iatrogenic root fracture during extraction occurred in 27.6% of treated dogs and 9.9% of extraction sites. The rate of intraoperative root fractures in the present study was substantially lower than the frequency of retained root fragments reported in a previous study, in which all extraction procedures had initially been considered successful by the treating practitioners (57). This finding further highlights the importance of meticulous preoperative planning and careful execution of dental extractions to minimize the risk of intraoperative complications. Although removal of fractured tooth fragments required removal of > 75% of the alveolar bone in all cases, it was not associated with statistically significant increased odds of dehiscence.

Prolonged anaesthetic duration is a known risk factor for surgical site infection in various surgical procedures (58–60). However, in a study evaluating incidence of surgical site infection following oromaxillofacial oncologic surgery, only anaesthetic events lasting greater than 6 hours were significantly associated with development of infection (61). Therefore, no increased odds of dehiscence were expected in the present study in relation to anaesthetic duration. However, reducing anaesthesia time whenever feasible remains an important surgical goal. Similarly, intraoperative systolic arterial pressure (SAP) < 80 mmHg and the need for crystalloid bolus therapy in response to hypotension were not identified as factors contributing to dehiscence in this study. However, SAP was monitored noninvasively using Doppler ultrasonography, which represents a limitation. More precise intraoperative blood pressure monitoring, such as direct arterial blood pressure measurement, should be considered in future studies to better evaluate the risks of oral wound healing complications in association with hypotension.

Intraoperative hypothermia has previously been reported as a significant risk factor for surgical site infection in both animal and human studies, although the evidence in animals remains inconsistent (58, 62–64). To the authors’ knowledge, animal studies specifically evaluating the effect of intraoperative body temperature on oral wound healing are lacking. In the present study, higher minimum intraoperative temperature was significantly associated with higher odds of dehiscence. However, this finding should be interpreted with caution, as the association was no longer evident after adjustment for body weight, which was also associated with increased dehiscence rates.

## 5 Conclusion

In summary, the incidence of wound dehiscence at 10 days following tooth extraction (without oronasal fistula and without any antibiotic and antiseptic treatment included) in healthy adult dogs was low when assessed at the level of individual extraction sites. However, when evaluated per treated dog, it remains a clinically relevant complication, that should be thoroughly discussed with owners. Notably, incidence of the dehiscence may have varied if an earlier or later re-check time was selected and/or re-check was performed on sedated dogs to allow for a more thorough assessment of surgical sites. Most dehisced sites healed by second intention and without the need for prolonged analgesic and/or antibiotic therapy, which is important when considering future management strategies for extraction wound dehiscence. None of the affected sites required surgical revision, and all sites healed spontaneously within one month after the procedure. Extraction of larger, periodontally healthy teeth in heavier dogs was associated with higher dehiscence rates. Other factors associated with a higher rate of wound dehiscence after exodontia included extraction sites in the rostral mandibular region, more extensive alveolar bone removal, and open extraction with a triangular flap. These factors are closely related to more complicated extractions and the resulting greater trauma to adjacent tissues. To the authors’ knowledge, this is the largest clinical prospective study to date evaluating the incidence of wound dehiscence after exodontia in healthy adult dogs and associated risk factors. As veterinary dentistry continues to grow and exodontia remains a commonly performed procedure, further studies are warranted. Future studies should ideally include more uniform canine populations and continue to evaluate different strategies to improve extraction wound healing.

## Data Availability

The original contributions presented in the study are included in the article/Supplementary material, further inquiries can be directed to the corresponding author.
