# Supplementary material for: Wound healing complications after tooth extraction in dogs
Source: Front Vet Sci. 2026 Oct 21;13:1962193. doi: 10.3389/fvets.2026.1962193 (PMC13730844; doi:10.3389/fvets.2026.1962193)
Supplement: Supplementary file 1 [file Table_1.DOCX]

**Supplementary material**

1. **POST-HOC PAIRWISE COMPARISONS**

Frequentist contrasts are shown as odds ratios (OR) with 95% confidence intervals and Tukey-adjusted p-values. Bayesian contrasts are shown as posterior median OR with 95% equal-tailed credible intervals and posterior probabilities for the direction of effect.

**Table S1.**
*Anatomical site pairwise comparisons*

| **Contrast** | | **OR** | **95% CI** | **Adjusted p** | |
| --- | --- | --- | --- | --- | --- |
| Cmand vs Inc | 2.82 | | 0.09–88.36 | | 0.924 |
| Cmax vs Inc | 4.65 | | 0.40–54.01 | | 0.428 |
| Rmand vs Inc | 23.25 | | 2.03–266.88 | | 0.004 |
| Rmax vs Inc | 6.84 | | 0.64–73.42 | | 0.176 |
| Cmax vs Cmand | 1.65 | | 0.07–37.60 | | 0.992 |
| Rmand vs Cmand | 8.25 | | 0.34–199.92 | | 0.370 |
| Rmax vs Cmand | 2.43 | | 0.11–53.26 | | 0.936 |
| Rmand vs Cmax | 5.00 | | 0.66–38.10 | | 0.194 |
| Rmax vs Cmax | 1.47 | | 0.21–10.36 | | 0.983 |
| Rmand vs Rmax | 3.40 | | 0.45–25.43 | | 0.460 |

**Table S2.**
*Bone removal pairwise comparisons*

| **Contrast** | **OR** | **95% CI** | **Adjusted p** |
| --- | --- | --- | --- |
| <50% vs None | 2.51 | 0.14–44.72 | 0.734 |
| ≥50% vs None | 18.92 | 1.57–227.83 | 0.016 |
| ≥50% vs <50% | 7.53 | 1.16–48.85 | 0.031 |

**Table S3.**
*Flap design Bayesian pairwise contrasts within open extractions only*

| **Contrast** | **OR** | **95% equal-tailed CrI** | **P(OR > 1)** |
| --- | --- | --- | --- |
| Pedicle vs Envelope | 6.19 | 0.24–79.40 | 0.878 |
| Triangle vs Envelope | 4.44 | 1.18–20.00 | 0.986 |
| Pedicle vs Triangle | 1.38 | 0.05–15.60 | 0.590 |

*Contrasts are derived from posterior draws from the Bayesian mixed-effects logistic regression restricted to open extractions. Envelope was the reference level in the fitted model.*

2. **CROSS-TABULATIONS OF SURGICAL FACTORS**

**Table S4A.**

*Flap type by extent of bone removal among open extractions*

| **Flap type** | **<50% bone removal** | **≥50% bone removal** | **Total** |
| --- | --- | --- | --- |
| Envelope | 45 (90.0%) | 5 (10.0%) | 50 |
| Pedicle | 2 (40.0%) | 3 (60.0%) | 5 |
| Triangle | 50 (38.8%) | 79 (61.2%) | 129 |
| Total | 97 | 87 | 184 |

*Data are presented as n (row %). Fisher’s exact test, p < .001.*

***Table S4B.***

*Intraoperative root fracture by extent of bone removal and dehiscence*

| **Intraoperative root fracture** | Bone removal | No dehiscence | Dehiscence | **Total** |
| --- | --- | --- | --- | --- |
| Absent | None | 210 | 1 (0.5 %) | 211 |
| Absent | <50% | 96 | 2 (2.0%) | 98 |
| Absent | ≥50% | 45 | 10 (18.2%) | 55 |
| Present | None | 0 | 0 | 0 |
| Present | <50% | 0 | 0 | 0 |
| Present | ≥50% | 36 | 4 (10.0%) | 40 |

*Data are presented as counts; values in parentheses indicate the proportion of extraction sites with dehiscence within each row.*

3. **POSTERIOR PARAMETER SUMMARIES**

**Table S5A.**
*Posterior parameter summaries for the flap-type model*

| **Parameter** | **Median** | **SD** | **95% equal-tailed CrI** |
| --- | --- | --- | --- |
| Intercept | -4.46 | 0.52 | -5.56 – -3.55 |
| Envelope vs None | -0.80 | 1.16 | -3.31 – 1.26 |
| Pedicle vs None | 2.59 | 1.54 | -0.84 – 5.15 |
| Triangle vs None | 2.15 | 0.55 | 1.12 – 3.28 |
| SD (dog intercept) | 0.18 | 0.79 | 0.00 – 2.79 |

*Posterior summaries are reported on the log-odds scale as posterior medians with equal-tailed 95% credible intervals. The intercept corresponds to the reference category (No flap).*

**Table S5B.**
*Posterior parameter summaries for the flap-type model restricted to open extractions*

| **Parameter** | **Median** | **SD** | **95% equal-tailed CrI** |
| --- | --- | --- | --- |
| Intercept | -3.84 | 0.71 | -5.38 – -2.60 |
| Pedicle vs Envelope | 1.82 | 1.49 | -1.44 – 4.37 |
| Triangle vs Envelope | 1.49 | 0.73 | 0.16 – 3.00 |
| SD (dog intercept) | 0.24 | 1.09 | 0.00 – 3.69 |

*Posterior summaries are reported on the log-odds scale as posterior medians with equal-tailed 95% credible intervals. The intercept corresponds to the reference category (Envelope flap).*
